# How should patient knowledge trigger clinical action in artificial intelligence-enabled pulmonary hypertension care?

**DOI:** 10.1093/ehjdh/ztag123

**Published:** 2026-07-25

**Authors:** Ilma Nascimento, Caio Julio Cesar dos Santos Fernandes

**Affiliations:** Pulmonary Circulation Unit, Instituto do Coração (InCor), Hospital das Clínicas, Faculdade de Medicina, Universidade de São Paulo, Av. Dr. Enéas Carvalho de Aguiar, 44, Cerqueira César, São Paulo 05403-900, Brazil; Pulmonary Circulation Unit, Instituto do Coração (InCor), Hospital das Clínicas, Faculdade de Medicina, Universidade de São Paulo, Av. Dr. Enéas Carvalho de Aguiar, 44, Cerqueira César, São Paulo 05403-900, Brazil

Armstrong and Winter provide a timely and persuasive account of how artificial intelligence (AI) may misrecognize patients by privileging what is readily measurable over what is most meaningful in lived illness.^1^ Their use of pulmonary hypertension (PH) is particularly valuable because this condition combines extensive physiological measurement with a burden that is only partly captured by conventional clinical endpoints. We agree that patients should be treated as epistemic partners rather than merely as sources of data. We suggest, however, that an additional implementation question deserves attention: what should happen after patient knowledge has been recognized?

In AI-enabled PH care, patient-reported outcome measures, free-text narratives, diaries, or conversational tools may identify worsening fatigue, reduced confidence, loss of independence, or increasing social restriction before clear changes appear in functional class, natriuretic peptides, or exercise capacity. Pulmonary hypertension-specific instruments such as emPHasis-10 and the Cambridge Pulmonary Hypertension Outcome Review (CAMPHOR) capture dimensions of symptoms, activity limitations, and quality of life that routine clinical measurements may not fully represent.^2,3^ Recognition of these signals is important, but their collection does not guarantee clinical consequence. Unless the care pathway defines who reviews the information, within what timeframe, according to which criteria, and with what authority to escalate care, patient knowledge may become visible without becoming actionable.

This distinction is especially important when patient-reported and physiological signals disagree. Deterioration in emPHasis-10 or CAMPHOR scores, or a narrative account of declining daily function, should not automatically trigger treatment escalation, because transient distress, comorbidities, measurement variability, and alert fatigue must also be considered. Conversely, apparent stability in conventional markers should not automatically override a consistent patient-reported decline. The clinically relevant task is therefore not simply to combine more data, but to establish a transparent process for interpreting discordance.

We propose that AI systems incorporating patient knowledge should be linked to a predefined response pathway with at least five elements: responsibility for reviewing the information; expected response time; criteria for confirmation or escalation; procedures for resolving disagreement between patient-reported and clinical measures; and communication back to the patient about what action, if any, followed. These elements should be co-designed with patients and caregivers before deployment, because acceptable thresholds and trade-offs may differ from those selected by clinicians or developers alone. Moreover, failure to engage with patient-generated information should not automatically be interpreted as non-adoption or disengagement by the patient; it may instead indicate that the technology has not been meaningfully integrated into the clinical workflow.^4^

Evaluation should also extend beyond model accuracy, fairness, and representativeness. Relevant implementation outcomes could include the proportion of patient-generated alerts reviewed, time from signal to clinical assessment, proportion leading to a documented action, reasons for non-action, patients’ perceptions of whether their concerns were addressed, and inequities in response according to digital access, geography, language, or socioeconomic position. Implementation frameworks distinguish successful adoption from effective and sustained incorporation into practice and may therefore help determine whether recognized patient knowledge actually influences care.^4,5^

Armstrong and Winter conclude that recognition is part of what makes a digital system clinically meaningful.^1^ We would extend this argument: recognition becomes clinically meaningful when it is connected to accountable interpretation and an observable response. As the authors develop the next phase of their work with patients and caregivers, do they envisage co-designing not only what AI should recognize, but also how recognized knowledge should change clinical pathways and how that change should be audited?
